# Lnc-PSMA8-1 activated by GEFT promotes rhabdomyosarcoma progression via upregulation of mTOR expression by sponging miR-144-3p

**DOI:** 10.1186/s12885-023-11798-y

**Published:** 2024-01-15

**Authors:** Lian Meng, Hao Shang, Qianqian Liu, Zhenzhen Li, Xiaomeng Wang, Qianru Li, Feng Li, Zhenguo Zhao, Chunxia Liu

**Affiliations:** 1 Department of Pathology and Key Laboratory for Xinjiang Endemic and Ethnic Diseases, Shihezi University School of Medicine/The First Affiliated Hospital, Shihezi University, Shihezi, China; 2 Department of Pathology, The Second Affiliated Hospital of Guangzhou Medical University, Guangzhou, China; 3 Department of Pathology and Medical Research Center, Beijing Chaoyang Hospital, Capital Medical University, Beijing, China; 4 Department of Orthopedics, National Clinical Research Center for Cancer/Cancer Hospital, National Cancer Center, Chinese Academy of Medical Sciences and Peking Union Medical College, Beijing, China; 5 Judicial Appraisal Institute, Tongde Hospital of Zhejiang Province (Zhejiang Mental Health Center), Hangzhou, China

**Keywords:** Rhabdomyosarcoma, ceRNA, GEFT, lnc-PSMA8-1, miR-144-3p, mTOR

## Abstract

**Background:**

GEFT is a key regulator of tumorigenesis in rhabdomyosarcoma (RMS), and overexpression of GEFT is significantly correlated with distant metastasis, lymph node metastasis, and a poor prognosis, yet the underlying molecular mechanism is still poorly understood. This study aimed to investigate and validate the molecular mechanism of GEFT-activated lncRNAs in regulating mTOR expression to promote the progression of RMS.

**Methods:**

GEFT-regulated lncRNAs were identified through microarray analysis. The effects of GEFT-regulated lncRNAs on the proliferation, apoptosis, invasion, and migration of RMS cells were confirmed through cell functional experiments. The target miRNAs of GEFT-activated lncRNAs in the regulation of mTOR expression were predicted by bioinformatics analysis combined with quantitative real-time polymerase chain reaction (qRT–PCR) analysis. The expression of lnc-PSMA8-1, miR-144-3p, and mTOR was measured by qRT–PCR in RMS tissue samples and cell lines. The regulatory mechanisms of the lnc-PSMA8-1-miR-144-3p-mTOR signaling axis were verified by RNA-binding protein immunoprecipitation (RIP), a luciferase reporter assay, qRT–PCR analysis, Western blot analysis, and cell functional experiments.

**Results:**

The microarray-based analysis identified 31 differentially expressed lncRNAs (fold change > 2.0, *P* < 0.05). Silencing the 4 upregulated lncRNAs (lnc-CEACAM19-1, lnc-VWCE-2, lnc-GPX7-1, and lnc-PSMA8-1) and overexpressing the downregulated lnc-FAM59A-1 inhibited the proliferation, invasion, and migration and induced the apoptosis of RMS cells. Among the factors analyzed, the expression of lnc-PSMA8-1, miR-144-3p, and mTOR in RMS tissue samples and cells was consistent with the correlations among their expression indicated by the lncRNA–miRNA–mRNA regulatory network based on the ceRNA hypothesis. lnc-PSMA8-1 promoted RMS progression by competitively binding to miR-144-3p to regulate mTOR expression.

**Conclusion:**

Our research demonstrated that lnc-PSMA8-1 was activated by GEFT and that the former positively regulated mTOR expression by sponging miR-144-3p to promote the progression of RMS. Therefore, targeting this network may constitute a potential therapeutic approach for the management of RMS.

**Supplementary Information:**

The online version contains supplementary material available at 10.1186/s12885-023-11798-y.

## Introduction

Rhabdomyosarcoma (RMS) is a type of soft tissue sarcoma observed occurring in children, which consists of skeletal myoblast-like cells with a high-grade neoplasm [1]. RMS can be divided into two histopathological subtypes: embryonal rhabdomyosarcoma (ERMS) and alveolar rhabdomyosarcoma (ARMS) [2]. The 5-year overall survival rates of children with RMS have improved significantly due to the adoption of multimodal therapeutic protocols [3]. However, children with high-risk RMS usually have low survival rates due to the development of chemoresistance and the metastasis and recurrence of this disease [4]. Uncovering the molecular mechanisms underlying RMS may assist in identifying novel therapeutic targets and improve the prognosis of patients with this malignancy.

Long noncoding RNAs (lncRNAs) are noncoding RNA constructs > 200 nucleotides in length that act as powerful intermediaries in numerous cellular physiological processes during the development and progression of almost all diseases [5]. Studies have shown that lncRNAs play roles in regulating cancer stem cells (CSCs) by targeting specific signaling pathways and transcription factors. The importance of lncRNAs as potential therapeutic targets for the elimination of CSCs has been emphasized in many research studies, as lncRNAs have the ability to maintain the characteristics of stem cells and facilitate the development of tumors by regulating gene expression. Some lncRNAs are well established to have tumor-specific expression, and these lncRNAs possess unique regulatory functions in tumor cells, ranging from mediating increases in invasion/migration to mediating recurrence, and have been considered prognostic/diagnostic biomarkers or therapeutic targets [6–9]. lncRNAs regulate tumor progression through a variety of mechanisms, and the function of lncRNAs as competitive endogenous RNAs (ceRNAs) allows them to abolish miRNA-mediated inhibition of target genes by sponging microRNAs (miRNAs/miRs) [10]. miRNAs are a type of endogenously activated small noncoding RNA, 18–25 nucleotides in length, that bind to the 3’-untranslated regions (3’-UTRs) of their target genes to regulate their expression. They reduce the stability and thus also the translational efficiency of mRNAs [11]. Therefore, the expression levels of tumor suppressors may be decreased and the expression levels of oncogenes increased by miRNAs during the initiation and/or development of RMS [12].

Guanine nucleotide exchange factor T (GEFT, ARHGEF25, or p63RhoGE), which is encoded by a gene located on chromosome 12q13.3, is a member of the Rho guanine nucleotide exchange factor family and is typically expressed in excitable tissues, including brain, muscle, and heart tissues. GEFT accelerates GDP/GTP exchange to activate Rho GTPases. It also plays essential roles in skeletal muscle regeneration and myogenic differentiation [13–16]. Our previous studies indicated that GEFT had high expression in RMS and that high GEFT expression was significantly related to poor prognosis, lymph node metastasis and distant metastasis [17, 18]. GEFT exerts its tumor-promoting effect via positive regulation of the proliferation, migration, invasion, and antiapoptotic capabilities of RMS cells via regulation of the Rac1/Cdc42-PAK signaling pathway to induce EMT [19]. mTOR is encoded by a gene located on chromosome 1p36.2 and is a member of the PI3K-related kinase family. It is often involved in regulating cell survival, growth, metabolism, protein synthesis, and autophagy, and the mTOR signaling pathway is dysregulated in numerous types of cancer and is frequently associated with carcinogenesis and tumor progression; thus, mTOR represents an ideal and promising therapeutic target. In addition, several studies have shown that lncRNAs are regulators of mTOR signaling in cancers [20]. In the present study, GEFT was found to positively regulate mTOR expression in RMS cells and to promote tumor progression to some extent through its ability to induce mTOR expression. However, the potential molecular mechanism by which GEFT modulates mTOR expression in RMS remains undetermined.

In the present study, a novel lncRNA, termed lnc-PSMA8-1 (ENST000000580975), was identified and shown to be activated by GEFT and highly overexpressed in RMS cell lines and tissues, which was indicative of poor prognosis. Next, it was shown that lnc-PSMA8-1 promoted the proliferation and migration of RMS cells and upregulated the expression of mTOR by sponging miR-144-3p. Thus, whether lnc-PSMA8-1, miR-144-3p, and/or mTOR could be considered novel therapeutic targets for RMS and how the lnc-PSMA8-1R/miR-144-3p/mTOR axis regulates RMS progression in vivo will be assessed in future studies.

## Materials and methods

### Clinical samples

In the present study, 20 paraffin-embedded RMS tissues and 10 normal skeletal muscle tissues were obtained from the First Affiliated Hospital, Shihezi University (Xinjiang, China) and the First Affiliated Hospital, Xinjiang Medical University (Xinjiang, China). The inclusion criteria were a diagnosis confirmed by two pathologists and the lack of systemic or local therapy prior to surgery. The exclusion criteria were a history of a second primary malignant tumor and local recurrence or metastasis. The pathological images of RMS samples are shown in Fig. S1. All the patients and their families were informed regarding specimen collection, and the patients’ parents/guardians provided written informed consent. All experiments were approved by the Ethics Committee of Shihezi University School of Medicine (No. 2019-021-01).

### Cell culture

The ERMS cell lines RD and A204 were obtained from the Cell Bank of the Chinese Academy of Sciences (Shanghai, China) and Fu Xiang Biotechnology Co., Ltd. (Shanghai, China). The ARMS cell lines RH30 and PLA802 were purchased from Shanghai Fu Xiang Biotechnology Co., Ltd. and Shanghai Hong Shun Biotechnology Co., Ltd. The human skeletal muscle cell line HSKMC was purchased from Beijing Be Na Biotechnology Co., Ltd. The above cell lines were cultured in DMEM (Gibco; Thermo Fisher Scientific, Inc.) supplemented with 10% fetal bovine serum (Biological Industries, Israel) and 1% streptomycin-penicillin (Solarbio, China) at 37 °C in a humidified incubator under a 5% CO_2_ atmosphere.

### Cell transfection

Shanghai GeneChem Co., Ltd. designed and synthesized the GEFT and lnc-FAM59A-1 overexpression plasmids, the GEFT interference plasmid, and the empty vector. The siRNAs against human lncRNAs (lnc-CEACAM19-1, lnc-VWCE-2, lnc-GPX7-1, lnc-PSMA8-1) and mTOR, the miR-144-3p mimic, the antisense miR-144-3p inhibitor, and the negative scramble control RNA oligo were purchased from Shanghai GenePharma Co., Ltd. Lipofectamine 2000 (Thermo Fisher Scientific, Inc.) was used for all transient transfections.

### RNA preparation and quantitative reverse transcription–PCR (qRT‒PCR)

Using a miRNeasy FFPE Kit or a miRNeasy Mini Kit (QIAGEN GmbH), total RNA was obtained from tissue samples or cultured cell lines, respectively. A Cytoplasmic and Nuclear RNA Purification Kit (Norgen Biotek Corp.) was used to isolate and purify cytoplasmic and nuclear RNA according to the manufacturer’s protocol. Reverse transcription was performed with a miScript II RT Kit (QIAGEN GmbH). cDNA was subsequently subjected to qRT‒PCR analysis on an Applied Biosystems 7500 Real-Time PCR System (Thermo Fisher Scientific, Inc.) using a miScript SYBR Green PCR Kit (QIAGEN GmbH). Subsequently, the samples were amplified by PCR, and the 2^-∆∆Ct^ method was used to calculate relative gene expression levels. The sequence-specific qRT‒PCR primers targeting miR-144-3p and U6 were designed and purchased from Shanghai GenePharma Co., Ltd. Additional RNA sequence-specific qRT‒PCR primers were acquired from Sangon (Shanghai, China). All of the sequences of the real-time PCR primers are listed in Table S1.

### Microarray analysis

Total RNA was obtained from GEFT-overexpressing and GEFT-knockdown RMS cells (RD, A204, RH30, and PLA802), amplified, and then used to synthesize fluorescent cRNA. The labeled cRNA was hybridized onto an Affymetrix Genechip® Human Transcriptome Array 2.0 (Affymetrix Inc.). The microarray experiments and data analyses were performed by Beijing Compass Biotechnology Co., Ltd.

### Cell proliferation and apoptosis assays

A CCK-8 assay (Dojindo Molecular Technologies, Inc.) was performed according to the manufacturer’s instructions to evaluate cell proliferation. Approximately 4 × 10^3^ cells per well were seeded into four 96-well plates and cultured with DMEM. After 0, 24, 48, or 72 h, 10 µl of CCK-8 reagent per well was added, and the cells were further incubated for 1.5 h at 37 °C. Subsequently, the optical density at 450 nm (OD450) was measured.

An Annexin V-APC/PI Apoptosis Detection Kit (KeyGEN, Chain) was used to analyze apoptosis 48 h post-transfection according to the manufacturer’s instructions. The apoptosis rate of cells was determined with a PAS flow cytometry system (PARTEC, Germany).

### Cell invasion and migration assays

A total of 2.5 × 10^5^ cells in 0.2 ml of serum-free DMEM were seeded into the upper chamber of a Transwell insert (8 μm pore size, Costar; Corning, Inc.) containing a membrane coated with Matrigel for the invasion assay and an uncoated membrane for the migration assay (BD Biosciences). After 24 h of incubation at 37 °C for 24 h, the transfected cells that had migrated through or invaded into the insert membrane were fixed and stained using 0.5% crystal violet solution. Subsequently, the number of invaded or migrated cells was determined using an optical microscope (Olympus BX51).

### RNA-binding protein immunoprecipitation (RIP)

A total of 5 × 10^5^ RD or RH30 cells were plated in 100 mm cell culture dishes and incubated for 24 h. Subsequently, the cells were transfected with the miR-144-3p mimic or miR-NC. Then, 48 h after transfection, in accordance with the manufacturer’s protocol, a RIP kit was used to assess the binding of endogenous Ago2 to RNA by RIP with an anti-Ago2 monoclonal antibody (Millipore Sigma); IgG was used as the control. Finally, the relative enrichment of lnc-PSMA8-1 and mTOR in the immunoprecipitates was determined by qRT‒PCR.

### Luciferase reporter assay

We used the DIANA, RNA22, miRanda and miRWalk2.0 bioinformatics tools to predict the binding sites between lnc-PSMA8-1 and miR-144-3p and between the mTOR 3’UTR and miR-144-3p. Luciferase plasmids that contained the wild-type lnc-PSMA8-1 binding site (lnc-PSMA8-1-WT) or mutated lnc-PSMA8-1 binding site (lnc-PSMA8-1-MUT) or the wild-type mTOR 3’UTR (mTOR 3’UTR-WT), or mutated mTOR 3’UTR (mTOR 3’UTR-MUT), the corresponding empty vector controls, and the Renilla luciferase plasmid were constructed by Shanghai GeneChem Co., Ltd. RD and RH30 cells were seeded into 24-well plates (3 × 10^4^ cells/well); 24 h later, the cells were transfected with 0.1 µl of one of the luciferase plasmids, 0.02 µl of the Renilla luciferase expression plasmid, and 100 nM miR-NC or the miR-144-3p-mimic. After 48 h, Renilla luciferase expression was measured according to the manufacturer’s protocol.

### Western blot analysis

Western blotting was used to measure the protein expression levels of mTOR and p-mTOR. Equal quantities of proteins were loaded into each lane of an SDS gel, separated using SDS‒PAGE, and transferred to PVDF membranes, which were then blocked with 5% BSA for 2 h. Since the positions where all protein blots appeared were quite stable and for obtaining clearer western blot bands, we set the upper and lower boundaries of the membranes according to protein molecular weight, and the left and right boundaries were according to different cell lines or other experiments. Therefore, all the blots were cropped.

prior to hybridization with primary antibodies. Subsequently, the membranes were incubated with primary antibodies overnight at 4 °C. The following antibodies were used: anti-β-actin (OriGene Technologies, Inc.), anti-mTOR, and anti-p-mTOR (both from Cell Signaling Technology, Inc.). Following six washes, the membranes were incubated with a secondary antibody (OriGene Technologies, Inc.) for a duration of 2 h and were then washed. Signals were visualized using chemiluminescence solution (Thermo Fisher Scientific, Inc.).

### Statistical analysis

SPSS 26.0 software was applied for statistical analysis. All data obtained from at least three separate experiments are presented as the means ± SDs. GraphPad Prism software was used to draw graphs. Differences with *P* < 0.05 were regarded as statistically significant (*P < 0.05, **P < 0.01, and ***P < 0.001).

## Results

### Screening of GEFT-regulated lncRNAs in RMS cell lines

**Fig. 1:**
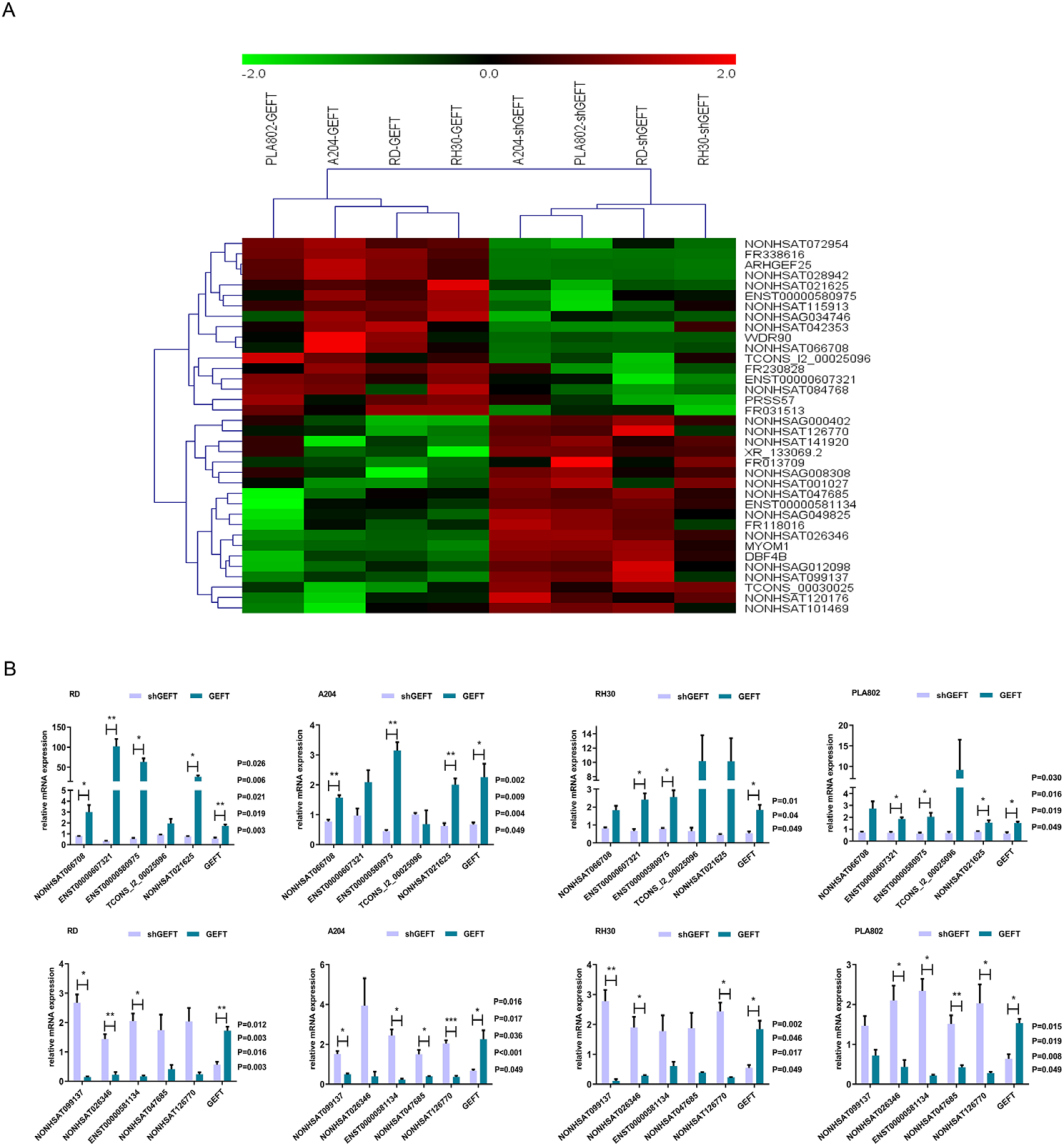
Screening of GEFT-regulated lncRNAs in RMS cell lines. **A:** Heat map representation of microarray data about the expression levels of GEFT-related lncRNAsand GEFT-related-mRNA in GEFT-overexpression and -knockdown RMS cell lines. **B:** Expression levels of randomly selected 5 up-regulated lncRNAs and 5 down-regulated lncRNAs in GEFT-overexpression and -knockdown RMS cell lines as detected by qRT-PCR

To identify the lncRNAs that are regulated by GEFT, microarray analysis was used, and lncRNA expression levels were compared between GEFT-overexpressing and knockdown cells. The microarray-based analysis identified 31 differentially expressed lncRNAs (*P* < 0.05, fold change > 2.0), namely, 14 upregulated lncRNAs and 17 downregulated lncRNAs (Fig. 1A, Table S2). To determine the reliability of the microarray chip analysis results, 10 lncRNAs were randomly selected (5 upregulated and 5 downregulated), and their expression was validated by qRT‒PCR. The results showed that the expression levels of these 10 lncRNAs were essentially consistent with the results of the microarray analysis (Fig. 1B).

### Effects of lnc-CEACAM19-1, lnc-VWCE-2, lnc-GPX7-1, lnc-PSMA8-1, and lnc-FAM59A-1 on the proliferation and apoptosis of RMS cells

**Fig. 2:**
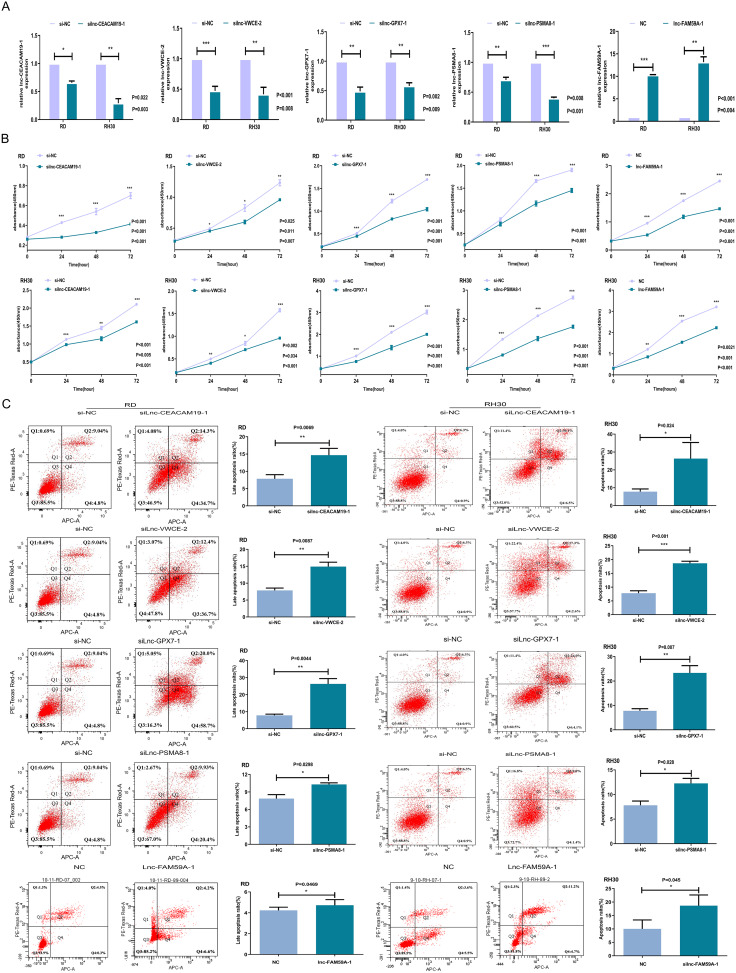
Effects of lnc-CEACAM19-1, lnc-VWCE-2, lnc-GPX7-1, lnc-PSMA8-1, and lnc-FAM59A-1 on the proliferation and apoptosis of RMS cells. **A**: Effects on RD and RH30 cells transfected with siRNA (lnc-CEACAM19-1, lnc-VWCE-2, lnc-GPX7-1, or lnc-PSMA8-1) or lnc-FAM59A-1 overexpressed plasmid as detected by qRT-PCR. **B**: Proliferative capabilities of RD and RH30 cells transfected with siRNA (lnc-CEACAM19-1, lnc-VWCE-2, lnc-GPX7-1, or lnc-PSMA8-1) or lnc-FAM59A-1 overexpressed plasmid as determined through CCK-8 cell proliferation assay. **C**: Apoptosis rates of RD and RH30 cells transfected with siRNA (lnc-CEACAM19-1, lnc-VWCE-2, lnc-GPX7-1, or lnc-PSMA8-1) or lnc-FAM59A-1 overexpressed plasmid as determined through flow cytometry assay

According to the microarray analysis and bioinformatic analysis results, four upregulated lncRNAs—namely, lnc-CEACAM19-1 (NONHSAT066708), lnc-VWCE-2 (NONHSAT021625), lnc-GPX7-1 (ENST00000607321), and lnc-PSMA8-1 (ENST00000580975)—and one downregulated lncRNA, lnc-FAM59A-1 (ENST00000581134), were predicted to regulate the biological functions of RMS cells. In RMS cell lines, we knocked down the upregulated lncRNAs (lnc-CEACAM19-1, lnc-VWCE-2, lnc-GPX7-1 and lnc-PSMA8-1) to assess their biological functions. Moreover, we overexpressed the downregulated lncRNA (lnc-FAM59A-1) to assess its biological functions. The qRT‒PCR results showed that the knockdown and overexpression were successful (Fig. 2A). Growth curves constructed using data from CCK-8 cell proliferation assays showed that knockdown of the four upregulated lncRNAs (lnc-CEACAM19-1, lnc-VWCE-2, lnc-GPX7-1 and lnc-PSMA8-1) and overexpression of the downregulated lnc-FAM59A-1 decreased the cell proliferation rate (Fig. 2B), and flow cytometric analysis showed that the late apoptosis rate in RD cells and total apoptosis rate in RH30 cells was also increased (Fig. 2C).

### Effects of lnc-CEACAM19-1, lnc-VWCE-2, lnc-GPX7-1, lnc-PSMA8-1, and lnc-FAM59A-1 on the invasive and migratory capacities of RMS cells

**Fig. 3:**
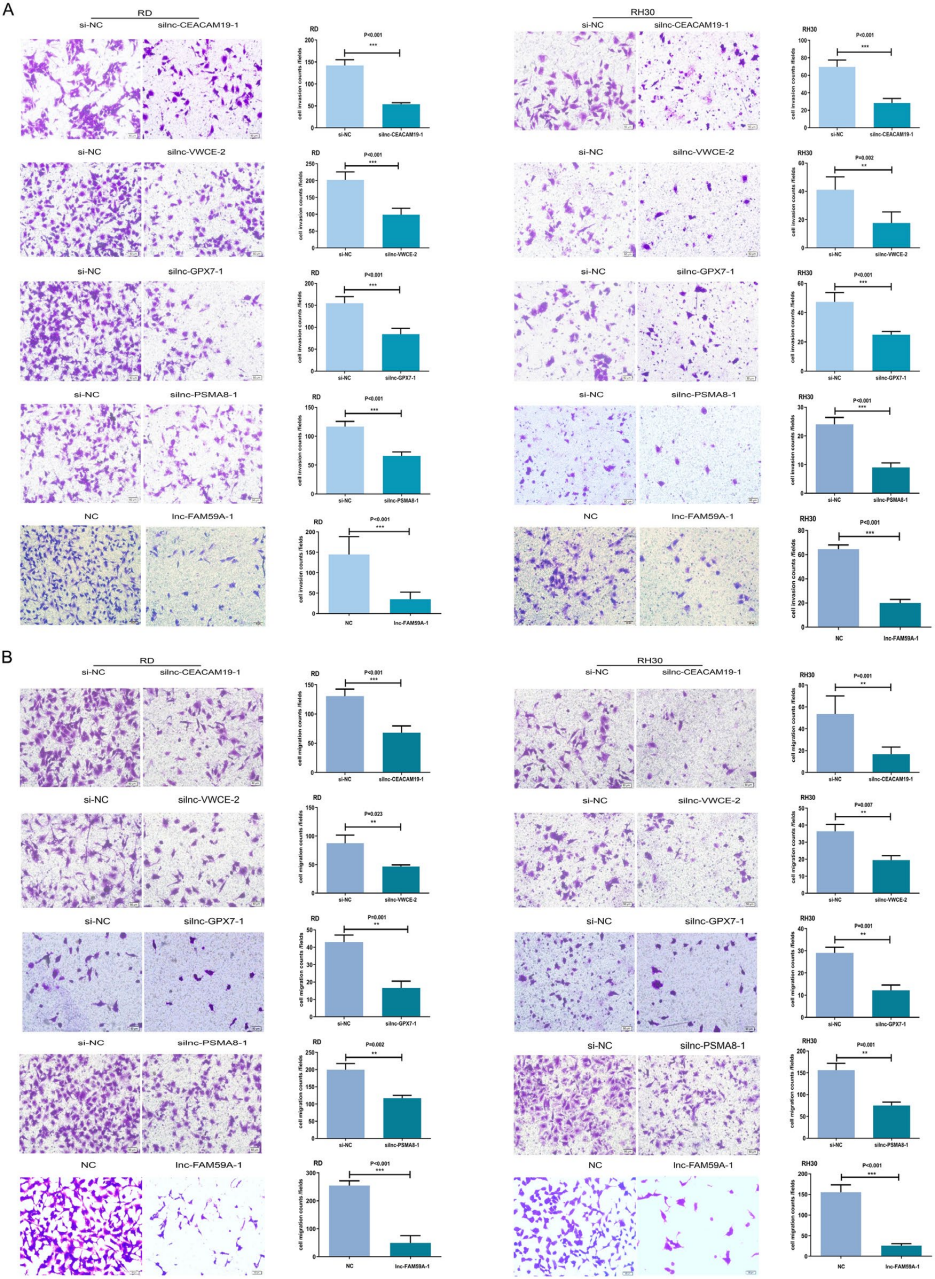
Effects of lnc-CEACAM19-1, lnc-VWCE-2, lnc-GPX7-1, lnc-PSMA8-1, and lnc-FAM59A-1 on the invasion and migratory of RMS cells.:**A**: Invasion of RD and RH30 cells transfected with siRNA (lnc-CEACAM19-1, lnc-VWCE-2, lnc-GPX7-1, or lnc-PSMA8-1) or lnc-FAM59A-1 overexpressed plasmid as determined through transwell system. **B**: Migratory of RD and RH30 cells transfected with siRNA (lnc-CEACAM19-1, lnc-VWCE-2, lnc-GPX7-1, or lnc-PSMA8-1) or lnc-FAM59A-1 overexpressed plasmid as determined through transwell system

The effects of lnc-CEACAM19-1, lnc-VWCE-2, lnc-GPX7-1, lnc-PSMA8-1, and lnc-FAM59A-1 on the invasion and migration of RMS cells were further studied using Transwell invasion and migration assays. The data revealed that knockdown of the four upregulated lncRNAs and overexpression of the downregulated lnc-FAM59A-1 reduced the invasion (Fig. 3A) and migration (Fig. 3B) of RMS cells.

### Mir-144-3p may play a bridging role between lnc-PSMA8-1 and mTOR

**Fig. 4:**
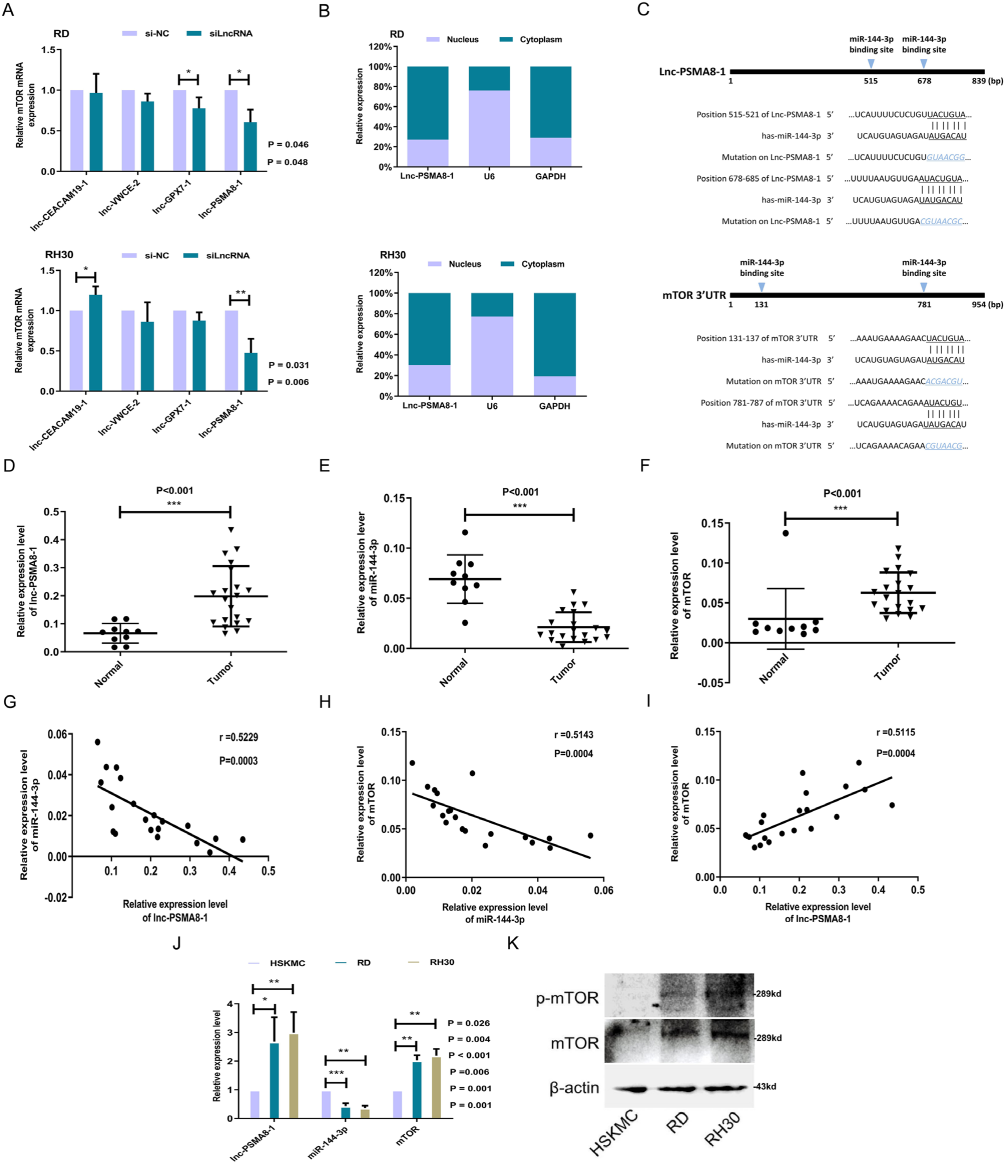
miR-144-3p may play a bridging role between lnc-PSMA8-1 and mTOR. **A**: Expression of mTOR in RD and RH30 cells transfected with siRNA (lnc-CEACAM19-1, lnc-VWCE-2, lnc-GPX7-1, or lnc-PSMA8-1) as detected by qRT-PCR. **B**: Lnc-PSMA8-1 expression in the nuclear and cytoplasmic RNA fractions obtained from RD and RH30 cells as shown by qRT-PCR. **C**: Predicted miR-144-3p binding sites in lnc-PSMA8-1 sequence and 3’-UTR of GEFT sequence and their induced mutations. **D-F**: Expression of lnc-PSMA8-1 **(D)** miR-144-3p (E) and mTOR **(F)** were detected in RMS samples (*n* = 20) and normal skeletal muscle samples (*n* = 10) by qRT-PCR. **G-I**: Correlation between lnc-PSMA8-1 and miR-144-3p, between miR-144-3p and mTOR, and between lnc-PSMA8-1 and mTOR as analyzed in 20 cases of RMS. **J**: Expression levels of lnc-PSMA8-1, miR-144-3p and mTOR in RD and RH30 cell lines as determined by qRT-PCR. **K**: Protein levels of mTOR and p-mTOR in RD and RH30 cell lines as determined by Western blot analysis. All the blots were cropped prior to hybridization with primary antibodies. The original blots are presented in Fig. S2

Accumulating evidence indicates that lncRNAs may serve as competitive endogenous RNAs (ceRNAs) to antagonize the functions of miRNAs; that is, lncRNAs sponge miRNAs to decrease their abundance and reduce their regulatory effects on their target 3’-UTRs. To examine whether the lncRNAs exert their specific effects on mediating the function of GEFT by functioning as ceRNAs to regulate mTOR expression in RMS cells, the upregulated lncRNAs that positively regulated mTOR expression in RD and RH30 cells were identified by transfection of siRNAs against lnc-CEACAM19-1, lnc-VWCE-2, lnc-GPX7-1, or lnc-PSMA8-1. As shown in Fig. 4A, interference with lnc-GPX7-1 and lnc-PSMA8-1 expression reduced the expression of mTOR in RD cells, but lnc-PSMA8-1 knockdown significantly decreased mTOR expression. Knockdown of lnc-PSMA8-1 notably reduced mTOR expression in RH30 cells, but this effect was not observed in RH30 cells transfected with siRNA targeting lnc-GPX7-1. Given the presence of miRNAs in the cytoplasm, the subcellular localization of lnc-PSMA8-1 was further determined. According to the results of analysis with the online bioinformatics tool lncLocator and qRT‒PCR, lnc-PSMA8-1 was localized primarily in the cytoplasm (Fig. 4B). Thus, lnc-PSMA8-1 possibly functions as a ceRNA that indirectly regulates mTOR expression. The DIANA and RNA22 online tools were used to identify miRNAs targeted by lnc-PSMA8-1, and miRanda and mirwalk2.0 were used to identify the miRNAs that target the 3’UTR of mTOR. The bioinformatics results combined with the results of whole-genome miRNA expression profiling in RMS cells indicated that miR-144-3p may play a bridging role between lnc-PSMA8-1 and mTOR (Fig. 4C).

Total RNA of RMS tissues and normal skeletal muscle tissues was extracted, and the expression of lnc-PSMA8-1, miR-144-3p, and mTOR was measured by qRT‒PCR to analyze their interrelationships. The results showed that the expression of lnc-PSMA8-1 and mTOR was higher in RMS tissues than in normal skeletal muscles and that the expression of miR-144-3p was lower in RMS tissues than in normal skeletal muscles (Fig. 4D-F). Spearman correlation analysis showed that miR-144-3p expression was negatively correlated with lnc-PSMA8-1 and mTOR expression and that lnc-PSMA8-1 expression was positively correlated with mTOR expression (Fig. 4G-I). Next, the expression levels of lnc-PSMA8-1, miR-144-3p, and mTOR were measured in human RMS and skeletal muscle cells. The results showed that the expression levels of lnc-PSMA8-1, miR-144-3p, and mTOR in these cells were identical to the levels measured in the corresponding tissues (Fig. 4J-K). These data suggested that the expression of lnc-PSMA8-1, miR-144-3p, and mTOR in RMS was consistent with the expression pattern determined based on the ceRNA-based regulatory lncRNA‒miRNA–mRNA network.

### lnc-PSMA8-1 modulates mTOR expression by competitively binding to miR-144-3p

**Fig. 5:**
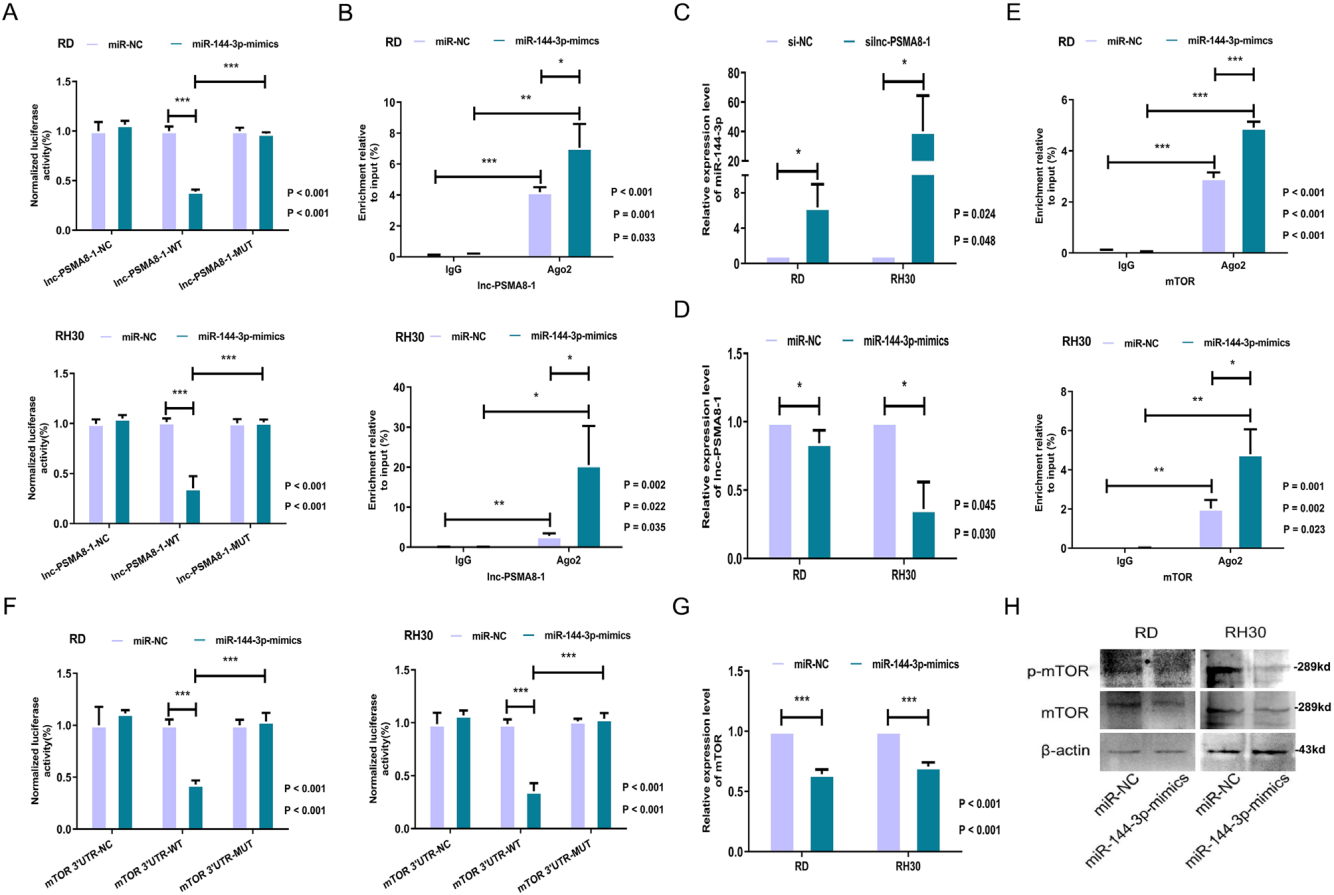
lnc-PSMA8-1 interacts with miR-144-3p and regulation of mTOR by miR-144-3p. **A:** RD and RH30 cells cotransfected with miR-144-3p mimics or negative control and luciferase reporter plasmid (lnc-PSMA8-1-WT/MUT/NC) were subjected to the luciferase reporter assay. **B:** Enrichment of lnc-PSMA8-1 bound to Ago2 or IgG in RD and RH30 cell lines as measured by qRT-PCR after RIP; IgG was used as a negative control. **C:** Expression levels of miR-144-3p in RD and RH30 cells transfected with lnc-PSMA8-1 siRNA or negative control as determined by qRT-PCR. **D:** Levels of lnc-PSMA8-1 in RD and RH30 cells transfected with miR-144-3p mimics or negative control as determined by qRT-PCR. **E:** Enrichment of mTOR bound to Ago2 or IgG in RD and RH30 cell lines as measured by qRT-PCR after RIP; IgG was used as a negative control. **F:** RD and RH30 cells cotransfected with miR-144-3p mimics or negative control and luciferase reporter plasmid (mTOR 3’UTR-WT/MUT/NC) were subjected to the luciferase reporter assay. **G:** Expression levels of mTOR in RD and RH30 cells transfected with miR-144-3p mimics or negative control as determined by qRT-PCR. **H:** Protein levels of mTOR and p-mTOR in RD and RH30 cells transfected with miR-144-3p mimics or negative control as determined by Western blot analysis. All the blots were cropped prior to hybridization with primary antibodies. The original blots are presented in Fig. S3 and Fig. S4

To determine the targeting effect of miR-144-3p on lnc-PSMA8-1, luciferase reporters containing the wild-type (lnc-PSMA8-1-WT) or mutated miR-144-3p binding site (lnc-PSMA8-1-MUT) were constructed. The results showed that overexpression of miR-144-3p reduced luciferase activity in cells transfected with the wild-type reporter vector but did not reduce luciferase activity in cells transfected with the mutant reporter vector or the empty vector (Fig. 5A). miRNAs bind to their target mRNAs, resulting in mRNA degradation and/or translational repression in a manner dependent on AGO2. For the purpose of ascertaining the regulatory effect of miR-144-3p on lnc-PSMA 8 − 1, anti-AGO2 RIP was performed in RD and Rh30 cells transiently overexpressing miR-144-3p. The AGO2-mediated endogenous lnc-PSMA8-1 precipitate exhibited significant enrichment in cells transfected with miR-144-3p (Fig. 5B). Moreover, knockdown of lnc-PSMA8-1 resulted in upregulated expression of miR-144-3p in RD and RH30 cells (Fig. 5C), whereas overexpression of miR-144-3p decreased lnc-PSMA8-1 expression in RD and RH30 cells (Fig. 5D). The data presented above support the hypothesis that lnc-PSMA8-1 acts as a ceRNA for miR-144-3p.

The bioinformatic analysis results revealed that mTOR may be a direct target of miR-144-3p. miRNA binds to its target mRNA and causes its posttranscriptional inhibition in an Ago2-dependent manner. Thus, anti-Ago2 RIP was used to confirm the association between mTOR and miR-144-3p. The results revealed that mTOR was notably enriched in the miR-144-3p precipitate, indicating that they are in the same RNA-induced silencing complex (Fig. 5E). To further validate the direct association between mTOR and miR-144-3p, luciferase reporters containing mTOR 3’UTR-WT or mTOR 3’UTR-MUT were constructed. Transient overexpression of miR-144-3p reduced luciferase activity in cells transfected with the wild-type reporter vector but not in those transfected with the mutant reporter or empty vector (Fig. 5F). In addition, qRT‒PCR and Western blotting showed that transient overexpression of miR-144-3p reduced mTOR expression (Fig. 5G-H). All these results indicated that miR-144-3p suppressed mTOR expression by directly targeting the 3’UTR of mTOR.

**Fig. 6:**
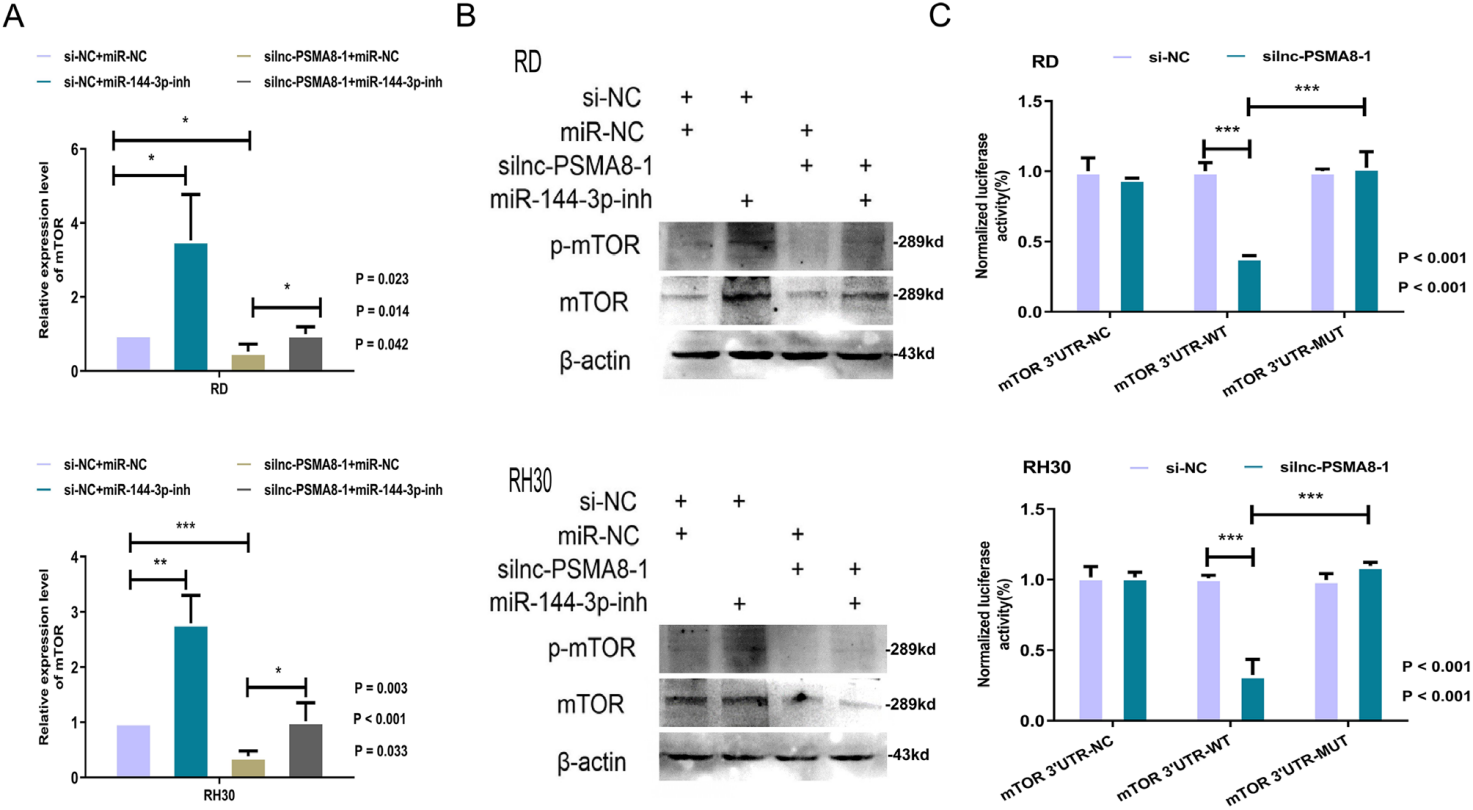
lnc-PSMA8-1 modulates mTOR expression via competitively binding to miR-144-3p. **A**: Expression of mTOR in RD and RH30 cells transfected with lnc-PSMA8-1 siRNA in combination with miR-144-3p inhibitor as detected by qRT-PCR. **B**: Protein levels of mTOR and p-mTOR in RD and RH30 cells transfected with lnc-PSMA8-1 siRNA in combination with miR-144-3p inhibitor as detected by Western blot analysis. **C**: RD and RH30 cells cotransfected with lnc-PSMA8-1 siRNA or negative control and luciferase reporter plasmid (mTOR 3’UTR-WT/MUT/NC) were subjected to the luciferase reporter assay. All the blots were cropped prior to hybridization with primary antibodies. The original blots are presented in Fig. S5

Since miR-144-3p is known to target mTOR for inhibition, can lnc-PSMA8-1 function through competitive binding to miR-144-3p, thereby attenuating the inhibitory effect of miR-144-3p on mTOR? To further investigate the role of lnc-PSMA8-1, the expression of mTOR in RD and RH30 cells was assessed after transfection of a siRNA to knock down lnc-PSMA8-1 combined with inhibitors of miR-144-3p. The results revealed that knockdown of lnc-PSMA8-1 decreased the expression of mTOR in RD and RH30 cells and that inhibition of miR-144-3p reversed the decrease in mTOR following the knockdown of lnc-PSMA8-1 (Fig. 6A-B).

The modulation of mTOR expression by miR-144-3p is dependent on the binding of miR-144-3p to the mTOR 3’UTR; if the regulatory effect of lnc-PSMA8-1 on mTOR is dependent on competitive binding of lnc-PSMA8-1 to miR-144-3p, then lnc-PSMA8-1 should also have a regulatory effect on the mTOR 3’UTR. Using the mTOR 3’UTR-WT and mTOR 3’UTR-MUT luciferase reporters, we found that depletion of lnc-PSMA8-1 decreased luciferase activity in cells transfected with mTOR 3’UTR-WT but not in cells transfected with the empty mutant reporter (Fig. 6C). These results indicate that lnc-PSMA8-1 modulates mTOR expression in RMS cells by competitively binding to miR-144-3p.

### lnc-PSMA8-1 promotes RMS progression partly through sponging mir-144-3p to regulate mTOR expression

**Fig. 7:**
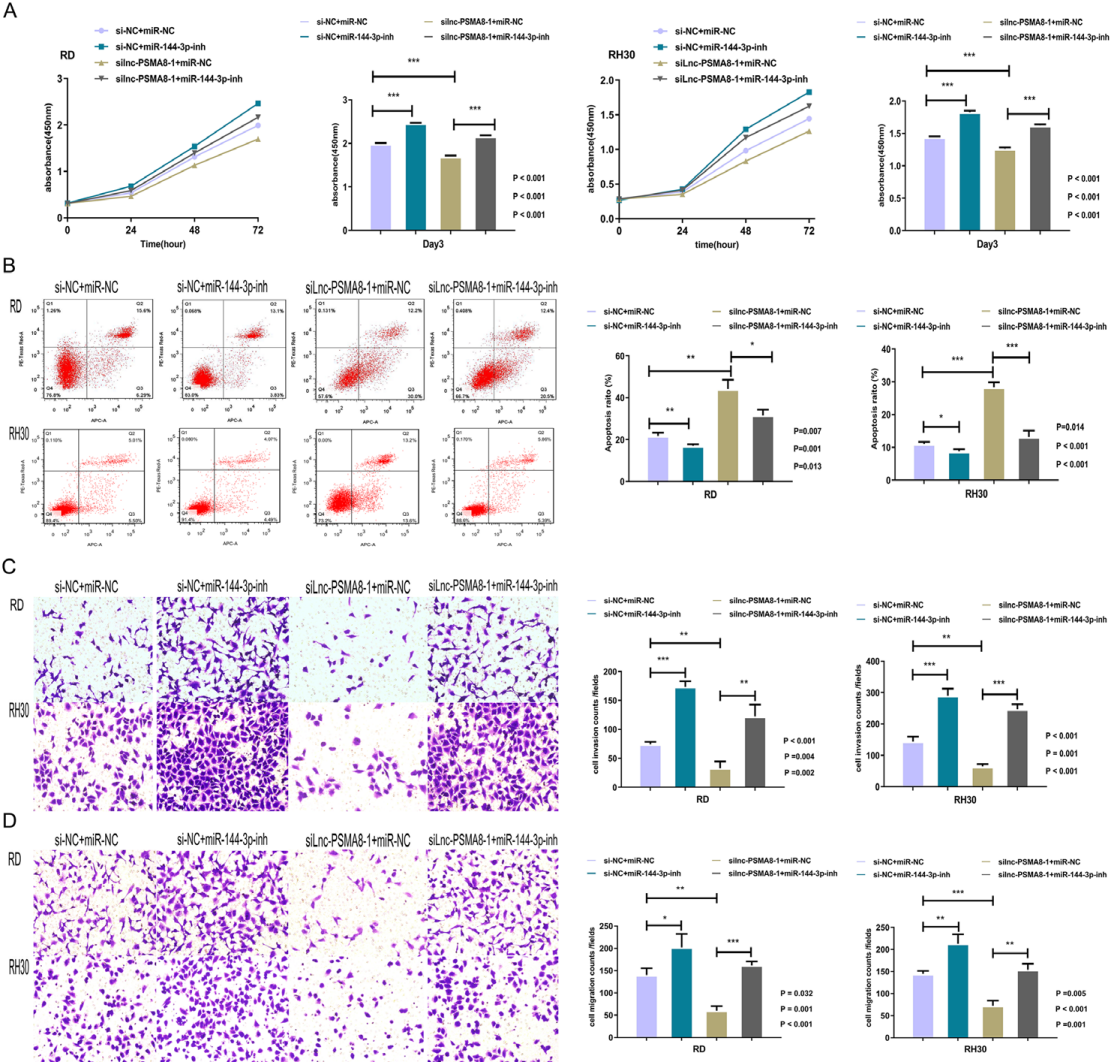
lnc-PSMA8-1 suppresses miR-144-3p function. **A:** Proliferative capability of RD and RH30 cells transfected with lnc-PSMA8-1 siRNA in combination with miR-144-3p inhibitor as determined through CCK-8 cell proliferation assay. **B:** Apoptosis rates of RD and RH30 cells transfected with lnc-PSMA8-1 siRNA in combination with miR-144-3p inhibitor as determined through flow cytometry assay. **C-D:** Invasion (C) and migratory (D) of RD and RH30 cells transfected with lnc-PSMA8-1 siRNA in combination with miR-144-3p inhibitor as determined through transwell system

To further confirm whether miR-144-3p affects the function of lnc-PSMA8-1 in RMS cells, the proliferative, apoptotic, invasive, and migratory capacities of RD and RH30 cells transfected with lnc-PSMA8-1 siRNA in combination with the miR-144-3p inhibitor were evaluated by a CCK-8 cell proliferation assay, flow cytometric analysis, and Transwell migration and invasion assays. Following lnc-PSMA8-1 knockdown, the proliferation of RMS cells was increased by miR-144-3p inhibition (Fig. 7A). Following knockdown of lnc-PSMA8-1, inhibition of miR-144-3p markedly reduced apoptosis (Fig. 7B). Moreover, inhibition of miR-144-3p reversed the suppressive effect of lnc-PSMA8-1 knockdown on the invasion (Fig. 7C) and migration (Fig. 7D) capabilities of cells. These observations suggest that lnc-PSMA8-1 promotes RMS progression by repressing miR-144-3p.

**Fig. 8:**
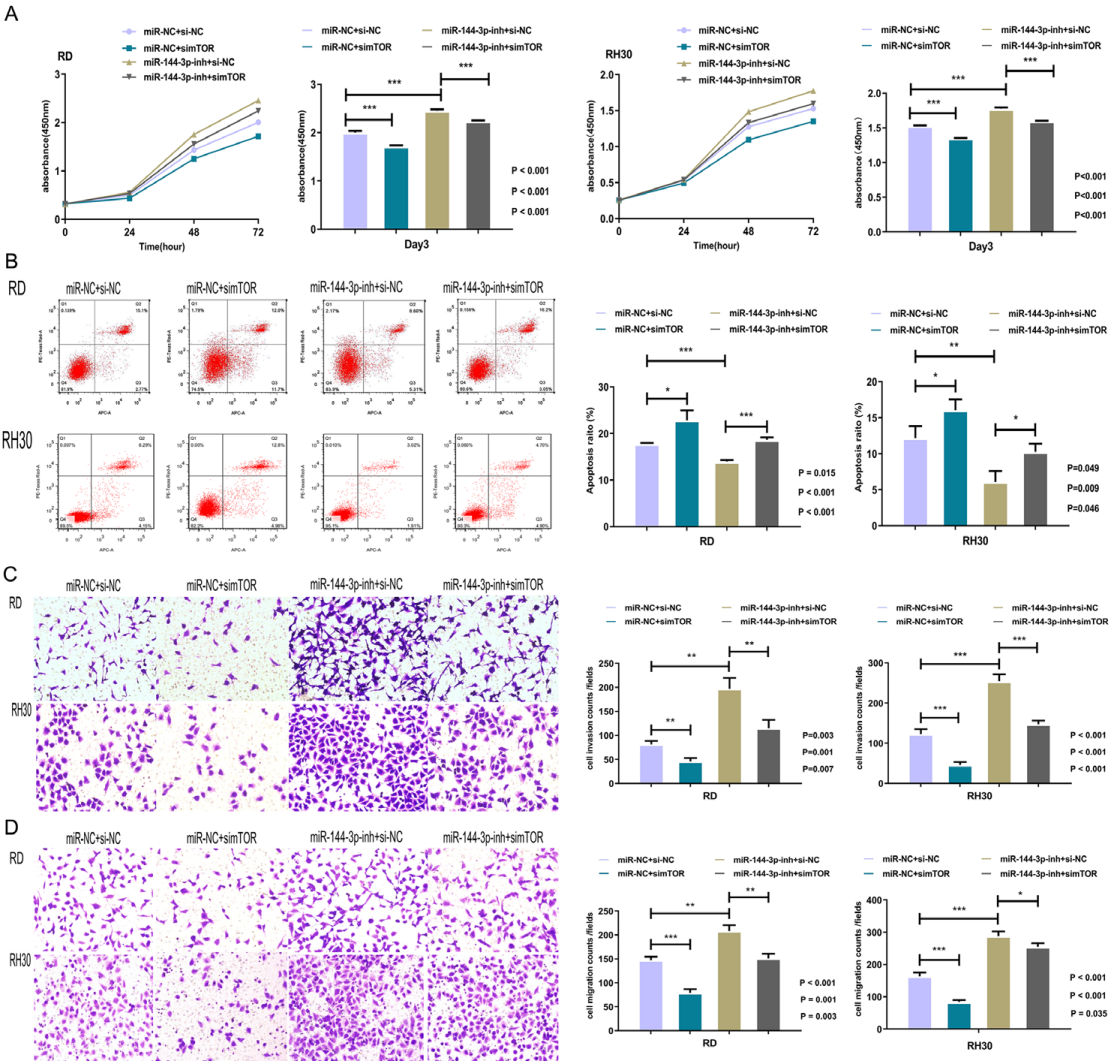
mTOR mediates the biological function of lnc-PSMA8-1-miR-144-3p axis. **A:** Proliferation of RMS cells in RD and RH30 cells transfected with miR-144-3p inhibitor in combination with mTOR siRNA as detected by CCK-8 cell proliferation assay. **B:** Apoptosis of RMS cells in RD and RH30 cells transfected with miR-144-3p inhibitor in combination with mTOR siRNA as detected by flow cytometry assay. **C-D:** Invasion (C) and migratory (D) of RMS cells in RD and RH30 cells transfected with miR-144-3p inhibitor in combination with mTOR siRNA as detected by transwell system

To investigate whether the modulatory effects of the lnc-PSMA8-1/miR-144-3p axis on the proliferative, apoptotic, invasive, and migratory capacities of RMS cells are mediated through the miR-144-3p target gene mTOR, these behaviors were evaluated in RD cells and RH30 cells transfected with the miR-144-3p inhibitor in combination with mTOR siRNA. Knockdown of mTOR inhibited the proliferation of RMS cells treated with the miR-144-3p inhibitor (Fig. 8A). Knockdown of mTOR reversed the reduction in apoptosis induced by the miR-144-3p inhibitor (Fig. 8B). Moreover, knockdown of mTOR markedly reduced the enhancing effects of miR-144-3p inhibition on the invasion (Fig. 8C) and migration (Fig. 8D) capabilities of cells. These results collectively suggest that lnc-PSMA8-1 promotes RMS progression by competitively binding to miR-144-3p to modulate mTOR expression.

## Discussion

Approximately 7% of all pediatric malignancies are soft tissue sarcomas, of which 50% of cases are RMS [3]. Radiation, combination chemotherapy, and surgery are commonly used approaches to treat RMS [21]. Over the past 50 years, several low-risk RMS patients have exhibited excellent outcomes, as shown by cooperative group trials [22]. Patients with distant metastases, who are at the greatest risk, have a maximum two-year event-free survival (EFS) rate of < 20% [23]. Despite a cooperative group trial being conducted in 1972, the outcome of those patients has not improved for five decades, highlighting the need to improve our understanding of the molecular mechanisms underlying this disease [24].

Approximately 2% of human genetic material encodes proteins, while the vast majority is transcribed into ncRNAs [25, 26]. Despite the annotation of thousands of lncRNAs in recent years, only a small fraction of them have undergone functional characterization [27].

ncRNAs can regulate chromatin function, influence the stability and translation of mRNAs within the cytoplasm, and interfere with signaling pathways through lncRNA–DNA, lncRNA–RNA, and lncRNA–protein interactions during pretranscriptional, transcriptional, or posttranscriptional processes [28]. It is possible that lncRNAs, which play crucial roles in almost all diseases, may eventually serve as therapeutic targets. There are theoretical advantages to this possibility. The high degree of specificity of lncRNA profiles across tissues and the regulation of cellular networks by lncRNAs suggests that targeting lncRNAs may have an advantage over targeting proteins in avoiding potentially harmful unintended consequences. Additionally, the lack of translation, fast degradation, and low expression levels of lncRNAs may allow more rapid effects with lower doses [10]. Therefore, searching for potential lncRNA therapeutic targets in rhabdomyosarcoma is a highly promising endeavor.

The GEFT gene is located on chromosome 12q13.3-24.1 and was validated to be overexpressed in RMS and associated with survival and prognosis. GEFT leads to metastasis and tumorigenicity in RMS by activating EMT induced by Rac1/Cdc42 signaling [17, 18, 29]. Here, microarray analysis was used to identify GEFT-regulated lncRNAs. In this study, knockdown of GEFT resulted in upregulation of lnc-CEACAM19-1, lnc-VWCE-2, lnc-GPX7-1, and lnc-PSMA8-1, and overexpression of GEFT resulted in downregulation of lnc-FAM59A-1, attenuating the malignant phenotypes of RMS cells. Then, it was found that lnc-PSMA8-1 was activated by GEFT and highly overexpressed in RMS cell lines and tissues, which was indicative of poor prognosis.

An increasing number of studies have shown that lncRNAs with multiple complementary miRNA binding sites function as ceRNAs or miRNA sponges, reducing miRNA function and indirectly targeting mRNAs, thus affecting the occurrence and development of tumors [30–33]. Wang et al. [34] demonstrated that the lncRNA HULC induced the phosphorylation of CREB by functioning as a ceRNA for miR-372 to reduce the translational repression of its target gene, PRKACB. Chen et al. [35] showed that LINC01234 in gastric cancer cells modulated CBFB expression by competitively binding to miR-204-5p. Yuan et al. [36] found that lncRNA-ATB was activated by TGF-β and accelerated hepatocellular carcinoma cell invasion by serving as a ceRNA for miR-200s to modulate the expression of ZEB1/2, ultimately inducing EMT.

Multiple types of cancer exhibit dysregulated mTOR signaling, and this pathway is frequently associated with carcinogenesis and tumor progression. According to reports, cancers with abnormal mTOR activation account for > 70% of all cases [37]. Therefore, targeting mTOR expression may serve as a novel strategy for the management of refractory RMS. lncRNAs can modulate mTOR activity in several ways as important modulators of mTOR signaling [20]. Thus, here, we examined whether the lncRNAs that regulate the role of GEFT also promote RMS progression by functioning as ceRNAs to regulate mTOR expression. We determined that lnc-PSMA8-1, one of the four GEFT-activated lncRNAs, positively regulated mTOR expression in RMS cell lines and was expressed mainly in the cytoplasm. According to the ceRNA hypothesis, lnc-PSMA8-1 possibly acts as a ceRNA to indirectly regulate mTOR expression. The bioinformatics results showed that miR-144-3p may play a bridging role between lnc-PSMA8-1 and mTOR.

Related studies have confirmed that multiple target sequences, including the 3’-UTR of mTOR, are regulated by miR-144-3p in several complex tumors. For example, Huo et al. [38] revealed that mTOR expression was downregulated by miR-144-3p in human salivary adenoid carcinoma, inhibiting cell proliferation and inducing apoptosis. Iwaya et al. [39] demonstrated that the progression of colorectal cancer was associated with the downregulation of miR-144, which targets mTOR. Ren et al. [40] revealed that miR-144 had a suppressive effect on the proliferation of osteosarcoma cells and induced apoptosis through the direct regulation of mTOR expression.

Hence, we assessed whether lnc-PSMA8-1, activated by GEFT, modulates mTOR expression by competitively binding to miR-144-3p to regulate biological behaviors of RMS cells. Our studies revealed that the expression of lnc-PSMA8-1, miR-144-3p, and mTOR in RMS tissues was consistent with the expression patterns suggested by a ceRNA-based lncRNA–miRNA–mRNA regulatory network. Mechanistic verification was also performed, which confirmed that lnc-PSMA8-1 modulated mTOR expression by competitively binding to miR-144-3p. The results of cell functional assays suggested that lnc-PSMA8-1 promoted cell proliferation, invasion, and migration and inhibited apoptosis in RMS cell lines through miR-144-3p via regulation of mTOR activity. These results collectively suggest that lnc-PSMA8-1 promotes RMS progression through competitively binding to miR-144-3p to regulate the expression of mTOR.

Notably, the of ceRNA hypothesis considers that all types of RNA transcripts interact via miRNA response elements [31]. Therefore, studies on lncRNAs acting as ceRNAs have primarily focused on the prediction and identification of lncRNA-targeted miRNAs. However, an often overlooked concept is that a ceRNA’s subcellular localization affects its accessibility to miRNAs. miRNAs are localized primarily in the cytoplasm, and lncRNAs can perform biological functions in the nucleus and in the cytoplasm [41–44]. lncRNAs with a nuclear localization typically control pretranscriptional or transcriptional processes. lncRNAs localized in the cytoplasm often act as ceRNAs that sponge miRNAs, thereby indirectly controlling the expression of target mRNAs at the posttranscriptional level [45]. Therefore, determining the subcellular localization of lncRNAs is necessary. The results of the bioinformatic analysis and cell fractionation assays in the present study confirmed that lnc-PSMA8-1 is localized primarily in the cytosol, and it regulates mTOR at the posttranscriptional level. In our study, we show that lnc-PSMA8-1 is an important modulator of mTOR.

## Conclusion

**Fig. 9:**
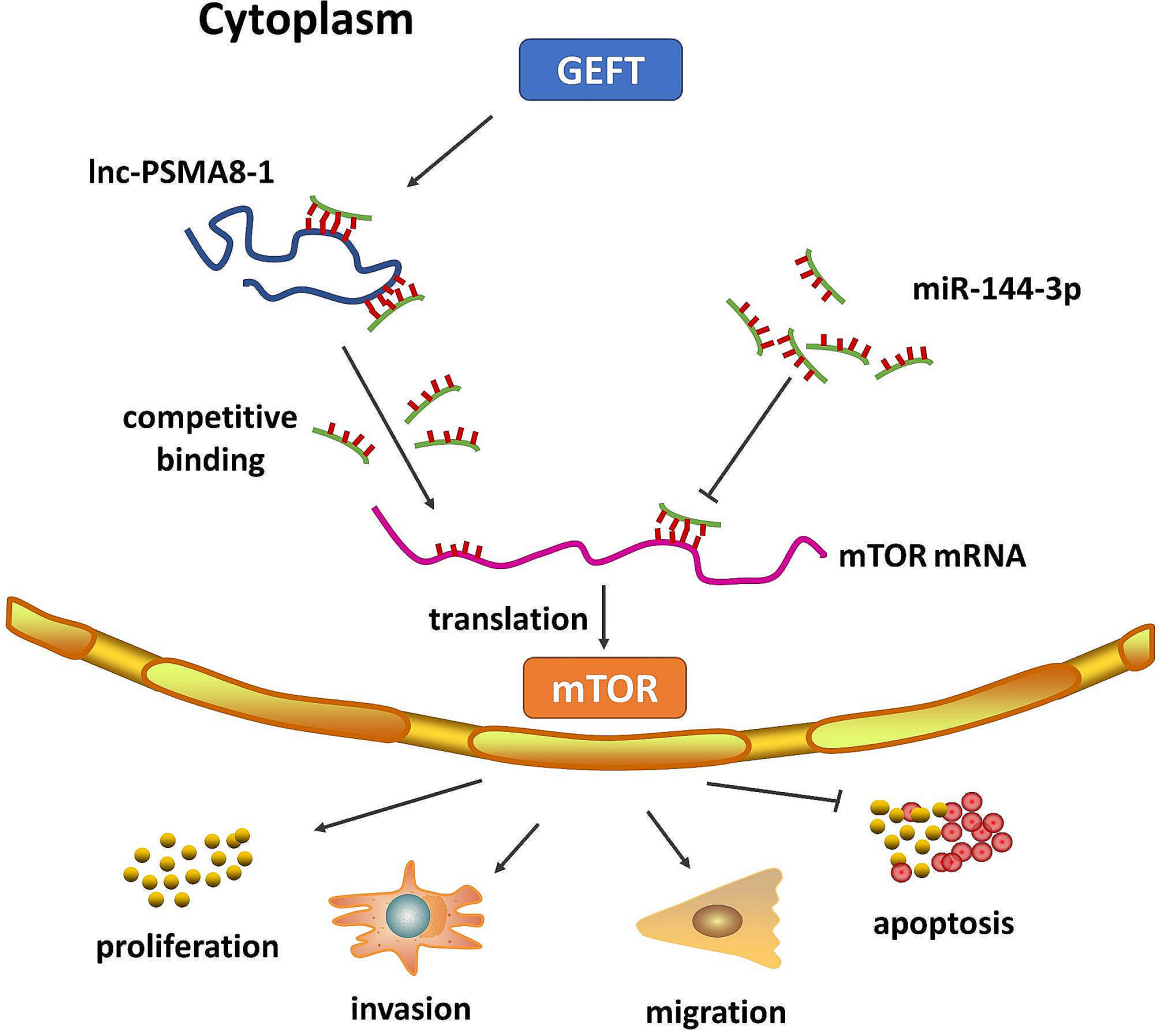
Lnc-PSMA8-1 activated by GEFT promotes RMS progression via acting as a ceRNA for miR-144-3p to regulate mTOR

Accordingly, our research demonstrated that lnc-PSMA8-1 is a key regulator of GEFT signaling pathways and that lnc-PSMA8-1, activated by GEFT, promotes RMS progression by functioning as a ceRNA of miR-144-3p to indirectly regulate the expression of mTOR. Thus, lnc-PSMA8-1 is activated by GEFT and promotes RMS progression by competitively binding to miR-144-3p to regulate the expression of mTOR (Fig. 9). lnc-PSMA8-1 could be an ideal and promising pharmacological target for therapeutic development in RMS.

### Electronic supplementary material

Below is the link to the electronic supplementary material.

Supplementary Material 1

Supplementary Material 2

Supplementary Material 3

## Data Availability

The original contributions presented in the study are included in the article/supplementary materials, further inquiries can be directed to the corresponding author.

## Abbreviations

ceRNA: Competitive endogenous RNA
EMT: Epithelial-mesenchymal transition
GEFT: Guanine nucleotide exchange factor T
lncRNA: Long noncoding RNA
mTOR: Mechanistic target of rapamycin kinase
qRT-PCR: Quantitative real-time polymerase chain reaction
RIP: RNA-Binding Protein Immunoprecipitation
RMS: Rhabdomyosarcoma
