# Supplementary material for: Lnc-PSMA8-1 activated by GEFT promotes rhabdomyosarcoma progression via upregulation of mTOR expression by sponging miR-144-3p
Source: BMC Cancer. 2024 Jan 15;24:79. doi: 10.1186/s12885-023-11798-y (PMC10789031; doi:10.1186/s12885-023-11798-y)
Supplement: Supplementary file 1 — Supplementary Material 1 [file 12885_2023_11798_MOESM1_ESM.docx]

**Table S1.** Primers of qRT-PCR.

| Gene | Primers |
| --- | --- |
| GEFT | F 5’-TAGGTACCACCATGCGGGGGGGGCACAAA-3’ |
|  | R 5’-CGACCGGTGACAGCTCATCTTCATCCAG-3’ |
| mTOR | F 5’-CGCTGTCATCCCTTTATCG-3’ |
|  | R 5’-ATGCTCAAACACCTCCACC-3’ |
| NONHSAT066708 | F 5’-AGCCAAAGACTCAGCAGCAC-3’ |
|  | R 5’-TTGAAGGAACAGACCGAAGG-3’ |
| ENST00000607321 | F 5’-ATTCAGAGGGCAGCATTGAC-3’ |
|  | R 5’-GAGGGGATAAGGGCAGAGAG-3’ |
| ENST00000580975 | F 5’-TCGAAGTGTTGGGATTACAGG-3’ |
|  | R 5’-AGCCTGGGTAACAAGAGCAA-3’ |
| TCONS_12_0025096 | F 5’-GCCATTGAGAAAGCCACCT-3’ |
|  | R 5’-GAAGGCAAAAGTCGGTCACT-3’ |
| NONHSAT021625 | F 5’-CTTCATCCTGGTTTGGACTCA-3’ |
|  | R 5’-AGATTTGGGCTGGAACGAC-3’ |
| NONHSAT099137 | F 5’-GTATTCCCCACCACCAAGAA-3’ |
|  | R 5’-ACCCACGCACAGAGTTTCA-3’ |
| NONHSAT026346 | F 5’-GAAAGAGAGGGAGGGAGAGAA-3’ |
|  | R 5’-GAACATTTGGGAAGAGAAGCA-3’ |
| ENST00000581134 | F 5’-CACCTCCTCTTGAATGCTCA-3’ |
|  | R 5’-GCCTCCCAAAGTGATGGA-3’ |
| NONHSAT047685 | F 5’-AGCCCTCCAATCTCCTGTCT-3’ |
|  | R 5’-TCTTTTGCTCCAGTGTGCTG-3’ |
| NONHSAT126770 | F 5’-GCGTTTGTCTTTGTTGTGGA-3’ |
|  | R 5’-ATGGATGGTAGGCAGAGGTC-3’ |
| β-Actin | F 5’-TCATGAAGTGTGACGTGGACAT-3’ |
|  | R 5’-CTCAGGAGGAGCAATGATCTTG-3’ |
| GAPDH | F 5’-ACCCAGAAGACTGTGGATGG-3’ |
|  | R 5’-TCTAGACGGCAGGTCAGGTC-3’ |
