# Supplementary material for: Lnc-PSMA8-1 activated by GEFT promotes rhabdomyosarcoma progression via upregulation of mTOR expression by sponging miR-144-3p
Source: BMC Cancer. 2024 Jan 15;24:79. doi: 10.1186/s12885-023-11798-y (PMC10789031; doi:10.1186/s12885-023-11798-y)
Supplement: Supplementary file 2 — Supplementary Material 2 [file 12885_2023_11798_MOESM2_ESM.docx]

**Table S2.** Microarray-based analysis found 31 differentially expressed lncRNAs, including 14 up-regulated lncRNAs and 17 down-regulated lncRNAs.

| TargetID | p-value | Fold-Change | Regulation |  |
| --- | --- | --- | --- | --- |
| FR338616 | 0.00000513 | 64.63934 | Up |  |
| ARHGEF25 | 0.000132 | 61.75424 | Up |  |
| NONHSAT028942 | 0.000125 | 58.9541 | Up |  |
| NONHSAT084768 | 0.03109149 | 3.370427 | Up |  |
| NONHSAT066708 | 0.025284363 | 3.157778 | Up |  |
| NONHSAT021625 | 0.006352586 | 2.9284563 | Up |  |
| ENST00000607321 | 0.012253516 | 2.8732364 | Up |  |
| NONHSAG034746 | 0.045264933 | 2.6320455 | Up |  |
| FR230828 | 0.028685078 | 2.3417163 | Up |  |
| TCONS_l2_00025096 | 0.04082119 | 2.2233112 | Up |  |
| ENST00000580975 | 0.031347588 | 2.2126355 | Up |  |
| NONHSAT072954 | 0.001056838 | 2.1781893 | Up |  |
| FR031513 | 0.008402912 | 2.1235664 | Up |  |
| NONHSAT042353 | 0.036932506 | 2.1121736 | Up |  |
| WDR90 | 0.0313824 | 2.0593305 | Up |  |
| PRSS57 | 0.028536765 | 2.0404475 | Up |  |
| NONHSAT115913 | 0.009280563 | 2.0164745 | Up |  |
| DBF4B | 0.001185424 | 12.94956 | Down | |
| MYOM1 | 0.000259 | 9.03684 | Down | |
| NONHSAT047685 | 0.024999417 | 5.33709 | Down | |
| NONHSAT099137 | 0.012741439 | 5.193948 | Down | |
| NONHSAT026346 | 0.000126 | 4.083355 | Down | |
| NONHSAT126770 | 0.038521 | 3.878599 | Down | |
| NONHSAG049825 | 0.015271372 | 3.4261162 | Down | |
| NONHSAT101469 | 0.041330237 | 3.342202 | Down | |
| TCONS_00030025 | 0.004878862 | 2.9667108 | Down | |
| NONHSAT001027 | 0.020728204 | 2.5537355 | Down | |
| NONHSAT120176 | 0.015000582 | 2.361651 | Down | |
| FR013709 | 0.038997464 | 2.284216 | Down | |
| ENST00000581134 | 0.039821718 | 2.2335007 | Down | |
| NONHSAG000402 | 0.012286826 | 2.1694808 | Down | |
| NONHSAT141920 | 0.029330818 | 2.1043856 | Down | |
| FR118016 | 0.040906053 | 2.0800781 | Down | |
| NONHSAG012098 | 0.009987087 | 2.033649 | Down | |
| NONHSAG008308 | 0.046046074 | 2.0201907 | Down | |
| XR_133069.2 | 0.026336933 | 2.0173447 | Down | |
