# Supplementary material for: Lnc-PSMA8-1 activated by GEFT promotes rhabdomyosarcoma progression via upregulation of mTOR expression by sponging miR-144-3p
Source: BMC Cancer. 2024 Jan 15;24:79. doi: 10.1186/s12885-023-11798-y (PMC10789031; doi:10.1186/s12885-023-11798-y)
Supplement: Supplementary file 3 — Supplementary Material 3 [file 12885_2023_11798_MOESM3_ESM.pdf]

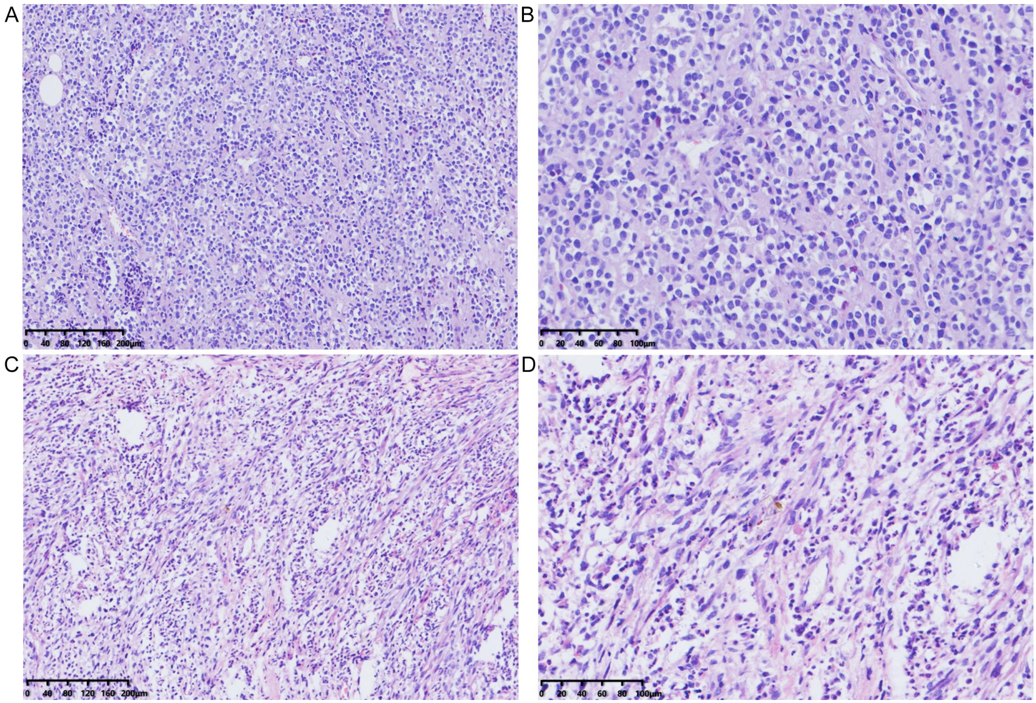

Fig. S1 Hematoxylin and eosin staining is shown for typical pathological image of rhabdomyosarcoma. A: ARMS, × 100; B: ARMS, × 200; C: ERMS, × 100; D: ERMS, × 200.

Fig. 4K

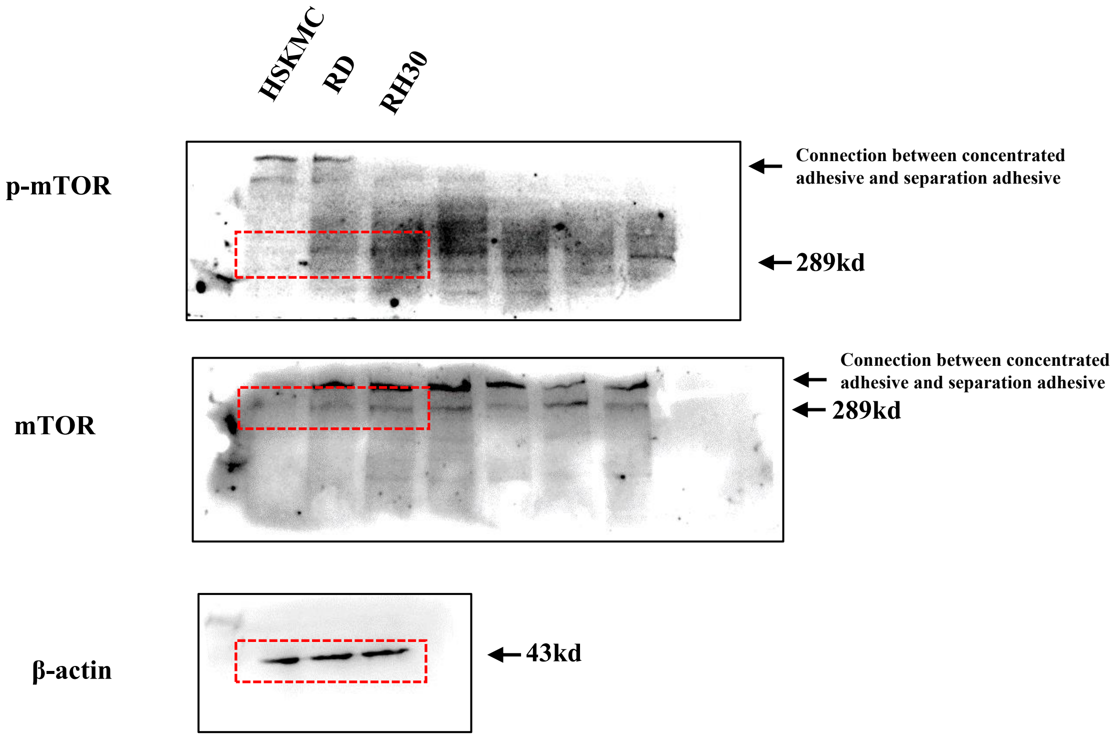

Fig. S2 The original images of western blotting in Figure 4K. Since the positions where the protein blots appeared were quite stable and for obtaining clearer bands, we set the upper and lower boundaries of the membranes according to protein molecular weight, and the left and right boundaries were according to different cell lines or other experiments. Therefore, all the blots were cropped prior to hybridization with primary antibodies. The red dashed boxes in the original blots indicate edges of membrane in Figure 4K of the manuscript.

Fig. 5H

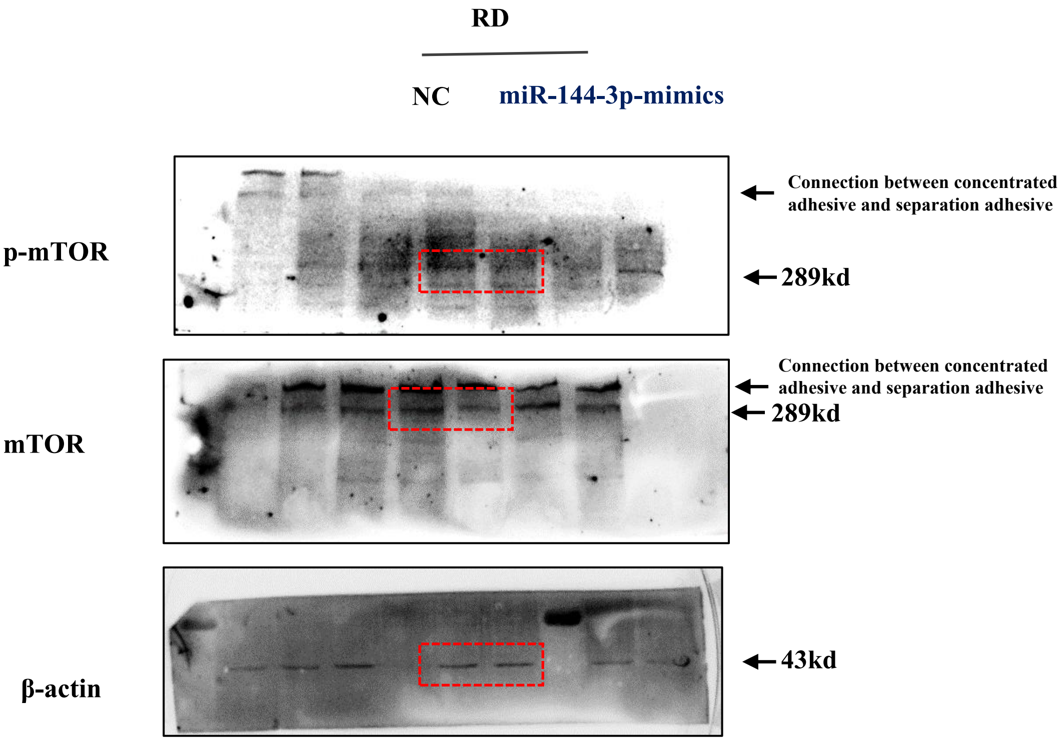

Fig. S3 The original images of western blotting in Figure 5H. Since the positions where the protein blots appeared were quite stable and for obtaining clearer bands, we set the upper and lower boundaries of the membranes according to protein molecular weight, and the left and right boundaries were according to different cell lines or other experiments. Therefore, all the blots were cropped prior to hybridization with primary antibodies. The red dashed boxes in the original blots indicate edges of membrane in Figure 5H of the manuscript.

Fig. 5H

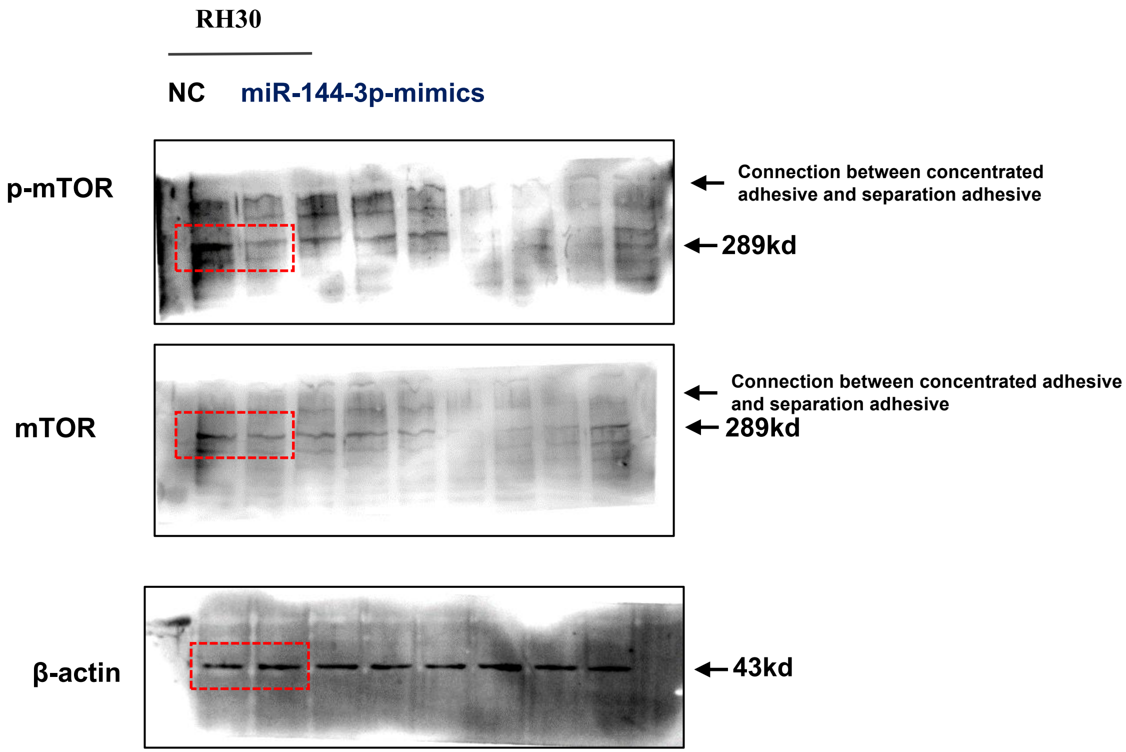

Fig. S4 The original images of western blotting in Figure 5H. Since the positions where the protein blots appeared were quite stable and for obtaining clearer bands, we set the upper and lower boundaries of the membranes according to protein molecular weight, and the left and right boundaries were according to different cell lines or other experiments. Therefore, all the blots were cropped prior to hybridization with primary antibodies. The red dashed boxes in the original blots indicate edges of membrane in Figure 5H of the manuscript.

Fig. 6B

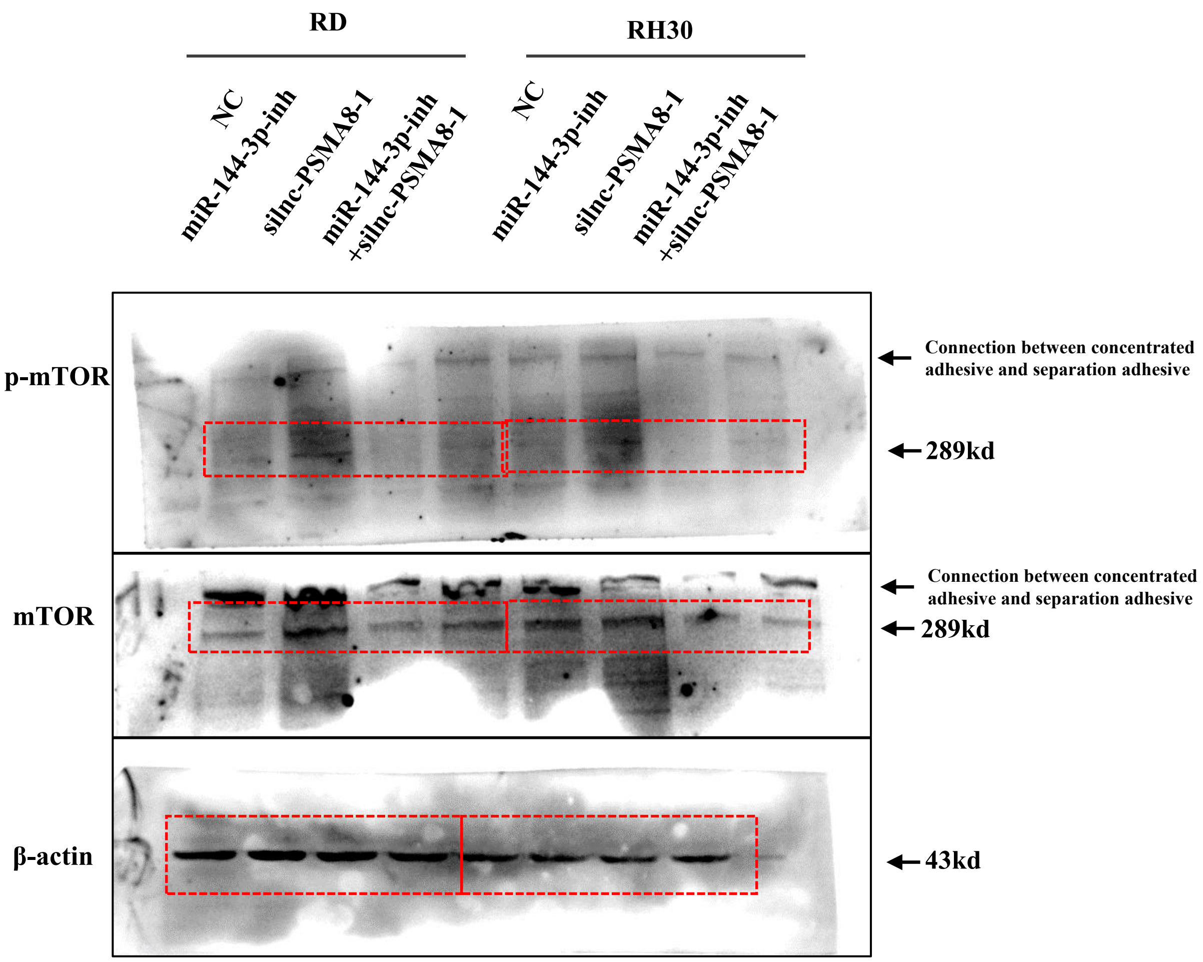

Fig. S5 The original images of western blotting in Figure 6B. Since the positions where the protein blots appeared were quite stable and for obtaining clearer bands, we set the upper and lower boundaries of the membranes according to protein molecular weight, and the left and right boundaries were according to different cell lines or other experiments. Therefore, all the blots were cropped prior to hybridization with primary antibodies. The red dashed boxes in the original blots indicate edges of membrane in Figure 6B of the manuscript.
